# Tungsten Oxide-Mediated Photocatalytic Silver Enhancement in a QCM Immunosensor for Alpha-Fetoprotein Detection

**DOI:** 10.3390/bios15110728

**Published:** 2025-11-02

**Authors:** Han Sol Kim, Yu Gyeong Cho, Soo Suk Lee

**Affiliations:** Department of Pharmaceutical Engineering, Soonchunhyang University, Asan 31538, Republic of Korea; itig2000@sch.ac.kr (H.S.K.); dbcandy@sch.ac.kr (Y.G.C.)

**Keywords:** alpha-fetoprotein (AFP), sandwich immunoassay, quartz crystal microbalance (QCM) biosensor, tungsten oxide, photocatalytic silver enhancement

## Abstract

Accurate and early detection of alpha-fetoprotein (AFP) in human serum is essential for the diagnosis and monitoring of hepatocellular carcinoma and related diseases. In this study, we present a highly sensitive and reproducible quartz crystal microbalance (QCM) immunosensor for the quantitative detection of AFP. The detection strategy is based on a sandwich-type immunoassay coupled with a signal amplification method utilizing photocatalytic silver deposition on tungsten(IV) oxide (WO_3_) nanoparticles. Since QCM detects resonance frequency shifts induced by mass changes on the sensor surface, the silver-enhanced growth of WO_3_ nanoparticles enables significant signal amplification, allowing for precise mass-based quantification. Without amplification, the limit of detection (LOD) for AFP using the QCM immunosensor was 286 pg/mL, which was significantly improved to 43.7 pg/mL with photocatalytic silver staining. This approach markedly improves both sensitivity and reproducibility of the assay, offering a robust and efficient platform for clinical biomarker detection and early cancer diagnostics.

## 1. Introduction

Cancer biomarkers are molecular indicators that reflect the biological state of tumors, offering valuable insight into cancer development, progression, and therapeutic responsiveness [1]. In clinical oncology, biomarkers are essential tools for stratifying patients, predicting disease prognosis, and guiding personalized treatment strategies. The sensitivity and specificity of cancer biomarker detection can greatly influence the efficacy of diagnostic and therapeutic decisions [2]. Detection of cancer biomarkers enables early diagnosis, which significantly improves patient prognosis and survival rates. The sensitivity and specificity of cancer biomarker detection can have a significant impact on the efficacy of diagnostic and therapeutic decisions. Despite remarkable advances in cancer treatment, the lack of early and accurate detection methods remains a major challenge, highlighting the need for sensitive biomarker detection platforms [3].

Alpha-fetoprotein (AFP), a glycoprotein produced primarily in the fetal liver and yolk sac, is one of the most widely used biomarkers for hepatocellular carcinoma (HCC) and germ cell tumors (GCTs). Although elevated levels of AFP are associated with malignancies, their presence in small quantities in adult serum necessitates highly sensitive and selective detection methods for clinical application [4,5]. Conventional immunoassays, such as enzyme-linked immunosorbent assays (ELISAs), though widely used, often lack the sensitivity required for early-stage cancer detection or for samples with low AFP concentrations. Therefore, biosensing platforms with enhanced sensitivity and signal amplification capabilities are of growing importance [6,7,8].

Quartz crystal microbalance (QCM) biosensors have attracted significant attention as mass-sensitive transducers capable of label-free detection of biomolecular interactions. QCM sensors operate based on the piezoelectric properties of quartz crystals, where the resonance frequency shifts in response to changes in mass on the crystal surface, as described by the Sauerbrey equation. As QCM is capable of detecting real-time mass changes at the sub-nanogram level, it has found broad applications in immunosensing, DNA hybridization, and molecular interaction studies. However, the limited sensitivity of QCM, most notably at low analyte concentrations, has led to the widespread adoption of signal amplification approaches [9,10,11,12].

Recent advances in nanotechnology have opened new possibilities for improving biosensor performance. Among these, nanoparticle-mediated signal amplification has shown remarkable potential. Gold nanoparticles (AuNPs) have been widely employed to enhance the QCM signal by increasing the mass on the sensor surface through sandwich-type immunoassay configurations [13,14,15,16,17]. Furthermore, enhancement techniques such as gold staining have enabled even greater signal magnitudes by catalytically growing metal layers around nanoparticle cores. Building on this foundation, alternative approaches utilizing photocatalytic deposition of metals such as silver on nanoparticle surfaces are being investigated for greater signal amplification and cost-effectiveness.

In particular, tungsten(IV) oxide (WO_3_) nanoparticles have emerged as a promising photocatalytic material due to their tunable bandgap, chemical stability, and strong oxidative power under UV or visible light. When irradiated, WO_3_ generates electron-hole pairs capable of driving redox reactions, such as the reduction of metal ions (e.g., Ag^+^) into metallic deposits on the nanoparticle surface [18,19,20,21]. This photocatalytic property can be harnessed to amplify the detectable mass on QCM biosensors. By functionalizing WO_3_ nanoparticles with antibodies and exposing them to silver ions under UV light, a silver shell can be deposited onto the WO_3_ particles, resulting in a significant increase in overall mass and a corresponding frequency shift in QCM measurements. Although silver has a lower atomic density and mass than gold, which may result in a relatively smaller signal amplification effect, its photo-induced deposition eliminates the need for additional reducing agents, enabling a more controlled and environmentally benign process [22,23]. Importantly, WO_3_ nanoparticles offer a dual functionality, acting both as photocatalysts and as nanocarriers for antibody immobilization, thereby streamlining sensor design and enhancing overall assay performance.

In this study, we report on a novel QCM immunosensor for AFP detection that integrates photocatalytic signal amplification via silver deposition on WO_3_ nanoparticles. The biosensor is constructed by covalently attaching anti-AFP capture antibodies onto a 3-Glycidyloxypropyltrimethoxysilane (3-GPTMS)-functionalized surface, which promotes stable and efficient antibody binding. A sandwich-type immunoassay is then conducted using AFP-containing serum and detection antibodies conjugated to WO_3_ nanoparticles. Upon UV irradiation in the presence of Ag^+^, photocatalytic silver deposition occurs selectively on the nanoparticle surface, resulting in a marked increase in resonator mass and a corresponding resonance frequency shift. This photocatalytic amplification method represents a significant advancement in QCM biosensing, offering improved sensitivity and lowering detection limits for AFP. Our approach not only broadens the scope of nanoparticle-based signal enhancement strategies but also demonstrates the potential of integrating semiconductor photocatalysts into label-free biosensing platforms. Ultimately, this technology could be extended to other clinically relevant biomarkers, paving the way for broader applications in early disease diagnostics and point-of-care testing.

## 2. Materials and Methods

### 2.1. Reagent and Materials

Recombinant Human AFP (ab114216) and two anti-AFP monoclonal antibodies (ab242532 for capture, ab242780 for detector) were purchased from Abcam (Cambridge, UK). AFP-free serum (P61000) was also obtained from BioPacific (Emeryville, CA, USA). Tungsten(IV) oxide (WO_3_) nanopowder (<100 nm particle size) and silver nitrate (AgNO_3_) for photocatalytic silver deposition, 3-Glycidyloxypropyltrimethoxysilane (3-GPTMS) for surface modification were purchased from Sigma-Aldrich Chemical Co. (St. Louis, MO, USA) and used without further purification. Bovine serum albumin (BSA) and 10X phosphate-buffered saline (PBS, pH 7.4) was also purchased from Sigma-Aldrich Chemical Co. A 1X PBS solution was prepared by diluting 10X PBS with double-distilled water. All organic solvents were purchased from Samchun Chemical Co., Ltd. (Seoul, Republic of Korea). All aqueous solutions were prepared in double-distilled water from a Milli-Q water-purifying system (18 MΩ, Millipore, Bedford, MA, USA).

### 2.2. Design and Configuration of the QCM Measurement System

**Figure 1:**
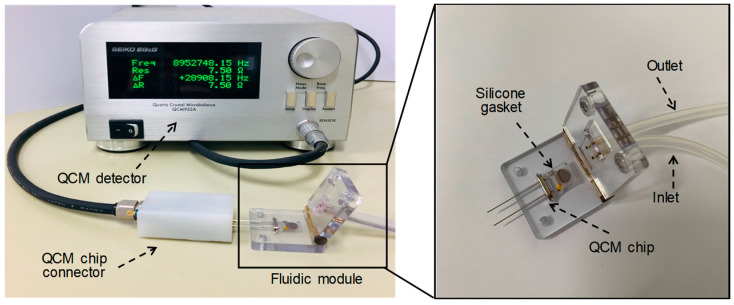
Schematic illustration of the QCM sensor integrated with custom-designed microfluidic modules.

The QCM measurement system consisted of fluidic and detection modules designed to provide real-time sensor data during assays. Mass changes on silicon dioxide-coated QCM resonators (5 mm diameter; 9 MHz resonance frequency) were monitored by tracking shifts in the resonance frequency of bulk acoustic waves using a QCM922A (Princeton Applied Research, SEIKO EG&G, Tokyo, Japan). The flow cell comprised a peristaltic pump (ISM597; ISMATEC, Glattbrugg, Switzerland), a custom-fabricated fluidic block, and a silicone rubber gasket. Precise fluid control is critical for reliable and user-friendly biomolecular detection in liquid environments. To address this, we developed a specialized fluidic module as shown in Figure 1. The top section of the fluidic block included recessed areas for reaction chambers and gaskets to prevent leakage caused by hydrodynamic pressure, along with integrated fluidic connectors enabling flow across the QCM sensors. Sample and buffer solutions were delivered to the reaction chambers via the peristaltic pump at a constant flow rate of 1.0 mL/min. Each reaction chamber had a volume of 20 µL. After each experiment, the chambers and gaskets were thoroughly rinsed with deionized water followed by 0.05% Tween 20 (Sigma-Aldrich, St. Louis, MO, USA) in PBS. Teflon^®^ tubing (0.032 in. I.D.; The Lee Company, Westbrook, CT, USA) was used to interconnect the fluidic and detection components.

### 2.3. X-Ray Diffraction (XRD) Analysis

Samples for XRD analysis were prepared by photocatalytic silver deposition on commercially available WO_3_ nanoparticles (1.5 × 10^−3^ M) dispersed in double-distilled water. The collected residues were oven-dried and analyzed using a Rigaku Miniflex 600 diffractometer (Wilmington, MA, USA). The XRD spectra were recorded in 2-thetha range between 5° and 90°.

### 2.4. Transmission Electron Microscopy (TEM) Characterization of WO_3_ and WO_3_/Ag Hybrid Nanostructures

For TEM imaging, highly diluted suspensions of WO_3_ nanoparticles (1.0 × 10^−5^ M) and WO_3_/Ag hybrid nanostructures were prepared in double-distilled water. These samples were sonicated for 2 h in an ultrasonic bath (Branson Ultrasonics Corp., Danbury, CT, USA) prior to imaging. TEM images were obtained using a JEM-1010 (JEOL, Tokyo, Japan) with an acceleration voltage of 80 kV.

### 2.5. UV–Vis Spectroscopic Analysis of Silver-Doped WO_3_ Nanostructures

To evaluate the photocatalytic activity of WO_3_ nanoparticles in the reduction of silver ions, the nanoparticles were dispersed in aqueous AgNO_3_ solutions with concentrations ranging from 0 to 20 mM. The mixtures were subjected to UV irradiation at a wavelength of 365 nm for durations ranging from 0 to 20 min using a handheld UV lamp. Upon irradiation, the solutions progressively developed a dark gray coloration, indicative of the formation of silver nanoparticles via photocatalytic reduction. The extent of silver nanoparticle generation was quantitatively assessed using UV–visible absorption spectroscopy. Absorbance measurements were performed in 96-well microplates (Thermo Fisher Scientific, Bremen, Germany) using an Epoch 2 microplate spectrophotometer (BioTek Instruments, Winooski, VT, USA). Each experimental condition was tested in quintuplicate to ensure reproducibility and statistical reliability.

### 2.6. Preparation of Antibody-Conjugated WO_3_ Nanoparticles for AFP Biosensing

WO_3_ nanoparticles (<100 nm particle size) were initially purified by sequential washing with methanol and deionized water, followed by drying in an oven at 80 °C. Surface activation was achieved by immersing the nanoparticles in 10 mL of freshly prepared piranha solution (H_2_SO_4_/H_2_O_2_ = 3:1, *v*/*v*) for 10 min. After thorough rinsing with deionized water, the activated nanoparticles were functionalized by treatment with 5% (*v*/*v*) 3-glycidyloxypropyltrimethoxy-silane (3-GPTMS) in methanol for 1 h at room temperature. The GPTMS-treated nanoparticles were subsequently washed with methanol (2 min), dried under a nitrogen (N_2_) stream, and cured at 110 °C for 1 h in a dry oven. Post-curing, the particles were again washed with methanol and dried under N_2_ to remove any unbound silane. For antibody conjugation, 10 μL of anti-AFP detecting antibody (1 mg/mL) was added to 1 mL of the GPTMS-modified WO_3_ nanoparticle suspension. The mixture was gently agitated at room temperature for 1 h to facilitate covalent binding. To block residual active sites and minimize nonspecific binding, 0.1 mL of 3% bovine serum albumin (BSA) in 1× phosphate-buffered saline (PBS, pH 7.4) was added, followed by incubation for 30 min at room temperature. The resulting antibody-conjugated WO_3_ nanoparticles were collected by centrifugation at 13,000 rpm for 10 min at 4 °C. After discarding the supernatant, the pellet was resuspended in 0.5 mL of 1 × PBS containing 0.1% BSA and stored for subsequent use.

### 2.7. WO_3_-Mediated Photocatalytic Silver Staining in Sandwich Immunoassay for AFP Detection

The SiO_2_-coated QCM sensor surface was first cleaned by sequential rinsing with deionized water and absolute ethanol, followed by nitrogen (N_2_) drying. The cleaned chip was then subjected to surface activation using UV/ozone treatment for 10 min. Subsequently, the activated surface was functionalized by immersion in 5% (*v*/*v*) 3-glycidyloxypropyl-trimethoxysilane (3-GPTMS) in methanol for 1 h at room temperature. Afterward, the chip was rinsed with methanol (2 min), dried under N_2_, and cured at 110 °C for 1 h. A final methanol rinse and N_2_ drying completed the silanization process. To immobilize the capture antibodies, 100 μg/mL of 30 μL of anti-AFP antibody solution (concentration adjusted for high-density immobilization, Figure S1) in PBS (pH 7.4) was deposited onto the GPTMS-modified QCM surface and incubated for 1 h in a humidity chamber to prevent evaporation. Unreacted surface sites were subsequently blocked with 3% bovine serum albumin (BSA) in PBS (pH 7.4) to minimize nonspecific binding during subsequent assay steps. In this blocking process, to minimize nonspecific adsorption during AFP detection using the QCM sensor, the BSA concentration in the blocking buffer was optimized, and 3% BSA was selected as it effectively reduced background signals (Table S1). Following surface preparation, 50 μL aliquots of AFP-spiked AFP-free human serum at various concentrations were applied to the sensor and incubated for 10 min to allow antigen binding. The surface was then washed with PBS (2 min), and 50 μL of WO_3_ nanoparticle-conjugated anti-AFP detecting antibody was introduced. After a further 10 min incubation, the chip was again washed with PBS (2 min). For signal amplification, 10 mM AgNO_3_ solution was added, and the sensor surface was irradiated with UV light (365 nm) using a 4 W handheld UV lamp (Vilber Lourmat, Marne-la-Vallée, France) for 5 min to initiate photocatalytic silver deposition mediated by the WO_3_ nanoparticles. The sensor was finally washed with PBS to remove excess reagents. All measurements were performed in triplicate for each AFP concentration, with independent replicates conducted on different days to ensure reproducibility. In addition, a blind test was conducted using five serum samples spiked with unknown concentrations of AFP. Each 1 mL sample was prepared using both the developed QCM sensor platform and the commercial VIDAS^®^ immunoassay system (BioMérieux, Marcy L’Étoile, France) to assess comparative performance.

## 3. Results and Discussion

### 3.1. Strategies for Sensitive AFP Immunoassay: Detection and Signal Amplification Approaches

**Scheme 1:**
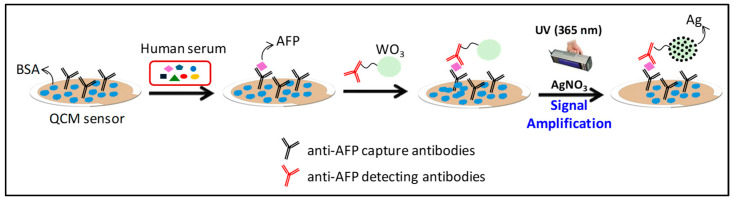
Schematic representation of the QCM-based AFP sandwich immunoassay integrated with WO_3_-mediated photocatalytic silver enhancement for signal amplification.

Scheme 1 presents the overall strategy for AFP detection, which is based on a conventional sandwich immunoassay format coupled with a photocatalytic signal amplification mechanism. In this approach, anti-AFP capture antibodies were covalently immobilized onto a QCM sensor surface pre-functionalized with 3-glycidyloxypropyltrimethoxysilane (3-GPTMS). Upon exposure to serum containing AFP, the target proteins specifically bound to the immobilized antibodies, forming the primary immune complex. Subsequently, detection antibodies conjugated to WO_3_ nanoparticles were introduced to form a complete sandwich structure. To amplify the sensor signal, the bound WO_3_ nanoparticles underwent a photocatalytic silver staining reaction, which increased their size and mass, thereby enhancing the resonance frequency shift measured by the QCM. This signal amplification was achieved through UV-induced reduction of silver ions (Ag^+^) on the WO_3_ nanoparticle surface, resulting in the formation of a WO_3_/Ag composite. The silver deposition step was critical for achieving sufficient signal intensity, as the resonance frequency shift without this amplification was negligible and inadequate for reliable AFP quantification. These results indicate that the majority of the observed frequency change during AFP detection originated from the photocatalytic silver staining process.

**Figure 2:**
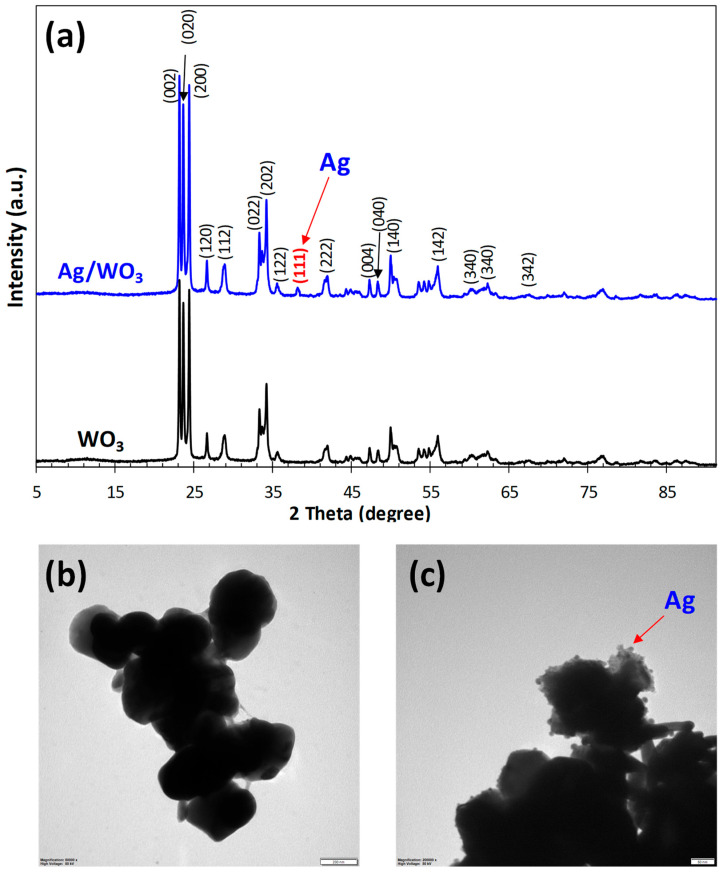
(**a**) XRD profiles of WO_3_ and Ag/WO_3_, highlighting changes in crystalline structure upon silver incorporation. Representative TEM images of (**b**) bare WO_3_ nanoparticles and (**c**) WO_3_/Ag nanocomposites were prepared using 10 mM AgNO_3_ and 5 min of UV irradiation.

To confirm the successful formation of the WO_3_/Ag composite, X-ray diffraction (XRD) analysis was conducted to investigate the crystal structures of WO_3_ and Ag-doped WO_3_ nanostructures, as well as to confirm the presence of silver particles on the WO_3_ surface following photocatalytic silver deposition. For comparison, the XRD pattern of pristine WO_3_ was also recorded. The diffraction results for both WO_3_ and Ag/WO_3_ specimens are presented in Figure 2a. As shown in Figure 2a, the typical diffraction spectrum of WO_3_ crystals exhibits the characteristic peaks of tungsten oxide. The XRD patterns of Ag-loaded WO_3_ clearly display the same set of peaks observed for pure WO_3_ (ICDD No. 43-1035) confirming the incorporation of silver into the tungsten oxide lattice. A distinct peak appearing at approximately 38.6° was identified as the (111) main reflection of metallic silver (Ag, ICDD No. 04-0783), which was absent in the pristine WO_3_ sample. These XRD results validate the successful fabrication of Ag/WO_3_ nanostructures through photocatalytic deposition of silver. Figure 2b,c show the representative TEM images of bare WO_3_ nanoparticles and WO_3_/Ag hybrid nanostructures formed by photocatalytic silver staining. In the latter, Ag nanoparticles are irregularly distributed on the WO_3_ surface, appearing as dark spots in the TEM image. Sample preparation for TEM imaging of bare WO_3_ and Ag-deposited WO_3_ was carried out separately in doubly distilled water.

### 3.2. Signal Amplification Process: WO_3_-Based Photocatalytic Silver Staining

**Figure 3:**
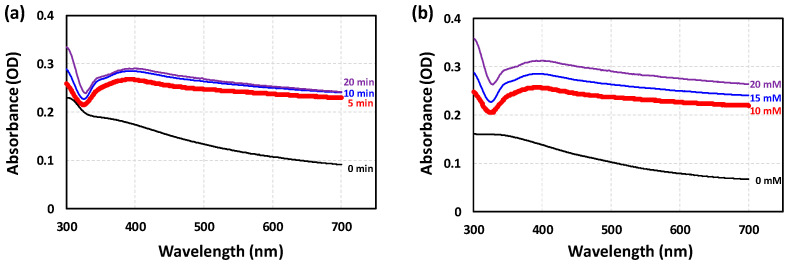
UV–VIS absorption spectra of WO_3_ nanoparticles: (**a**) dispersed in 10 mM AgNO_3_ solution under different UV irradiation times (0, 5, 10, and 20 min), and (**b**) suspensions after 5 min of UV irradiation in AgNO_3_ solutions with varying concentrations (0, 10, 15, and 20 mM).

To improve the detection sensitivity of the QCM-based sandwich immunoassay, we performed a photocatalytic silver staining reaction, which is crucial for amplifying the mass-induced resonance shift in the QCM sensor. Given that the silver staining process relies on UV-induced reduction of Ag^+^ ions in aqueous AgNO_3_ solution in the presence of WO_3_ nanoparticles, two critical factors were investigated: AgNO_3_ concentration and UV irradiation time. Throughout this process, the intensity of UV light was held constant to exclude its influence on reaction kinetics. First, the effect of UV irradiation duration was assessed by dispersing WO_3_ nanoparticles in 10 mM AgNO_3_ solution and monitoring the absorption band appeared in the visible region, which is a typical phenomenon associated with silver formation [24]. As shown in Figure 3a, the UV–vis spectra recorded at various time points revealed a progressive increase in absorbance at 400 nm with prolonged UV exposure. Based on the rate of absorbance increase and the overall immunoassay time for AFP detection, we determined that 5 min of UV irradiation was sufficient. Next, the influence of AgNO_3_ concentration was investigated under the fixed condition of 5 min UV exposure. As shown in Figure 3b, the absorbance at 400 nm increased with increasing AgNO_3_ concentration up to 20 mM. A concentration of 10 mM was considered sufficient to achieve uniform and stable silver deposition. At higher concentrations, excessive nucleation of silver could lead to uncontrolled nanoparticle growth and potential detachment from the WO_3_ surface. This was further evidenced in QCM experiments, where sensor instability and signal deterioration were observed due to silver nanoparticle aggregation and shedding from the surface (Figure S3). As shown in Figure S3, excessive AgNO_3_ concentrations (≥15 mM) induced aggregation and partial detachment of Ag nanoparticles, in contrast to the uniform deposition observed at 10 mM AgNO_3_ (Figure 2c). Based on these observations, the conditions for WO_3_-mediated photocatalytic silver staining were established as 10 mM AgNO_3_ and 5 min of UV irradiation. These parameters were employed in all subsequent signal amplification steps within the AFP sandwich immunoassay.

### 3.3. Real-Time QCM Sensor Response for AFP Detection

**Figure 4:**
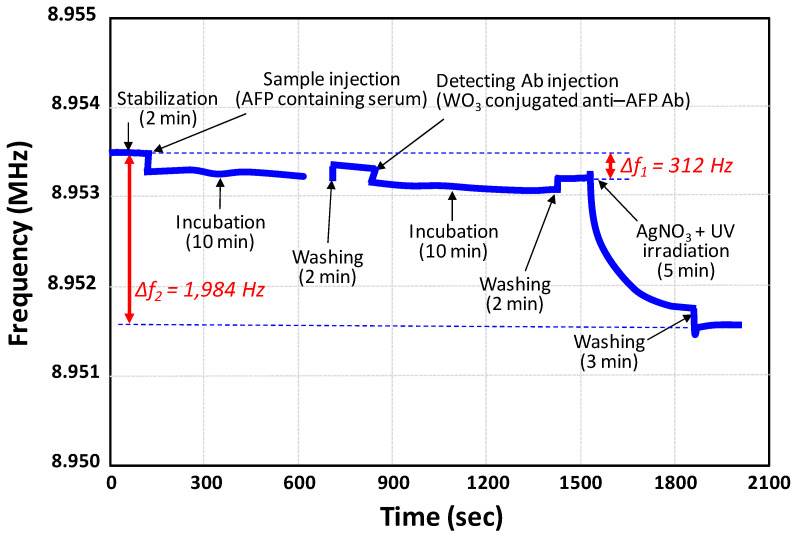
Real-time QCM sensor response to 10 ng/mL of AFP in human serum, detected via WO_3_-conjugated antibody sandwich immunoassay followed by photocatalytic silver staining for signal amplification. The observed decrease in resonance frequency corresponds to the increase in effective mass on the QCM sensor surface. The total frequency shift (*Δ* frequency) is indicated by the red double-headed arrow in the figure. *Δf_1_* and *Δf_2_* represent the frequency changes induced by the sandwich immunoassay alone and the subsequent photocatalytic silver deposition, respectively.

Figure 4 illustrates the real-time changes in resonance frequency and resistance of the QCM sensor during the stepwise detection of AFP. The sensing process comprises three main stages: (1) immobilization and binding of AFP to surface-anchored capture antibodies, (2) formation of sandwich complexes through the introduction of WO_3_-conjugated detection antibodies, and (3) signal amplification via photocatalytic silver staining, leading to size enhancement of the WO_3_ nanoparticles. The entire process takes approximately 30 min. As anticipated, the resonance frequency decreased progressively due to mass accumulation on the sensor surface during each sequential step, while the resonance resistance concurrently increased, reflecting energy dissipation associated with the growing viscoelastic layer (Figure S2) [25]. The blue bold line in Figure 4 represents the temporal decrease in resonance frequency observed during AFP detection at a concentration of 10 ng/mL. Notably, the most significant shifts in both parameters occurred during the photocatalytic silver deposition phase. This amplification step, in which metallic silver was selectively deposited onto the surface of WO_3_ nanoparticles, substantially increased the total mass bound to the sensor, resulting in a pronounced frequency drop. These findings underscore the critical role of the silver staining step in enhancing signal magnitude. In the absence of this step, the sensor exhibits a markedly weaker response, thereby compromising detection sensitivity. Thus, the incorporation of photocatalytic silver amplification is essential for achieving ultrasensitive AFP detection using the QCM platform. The substantial increase in mass observed on the QCM sensor surface following the photocatalytic deposition of metallic silver onto WO_3_ nanoparticles resulted in a pronounced decrease in resonance frequency, as described by the Sauerbrey equation (Equation (1)) [26]:

(1) ∆f=−2f02/Aρq×μq12

where *f_0_* is the fundamental resonance frequency (8.953 × 10^6^ Hz), *Δf* is the change in frequency (Hz), *Δm* is the change in mass (g), A is the active area of the piezoelectric quartz crystal (0.196 cm^2^), and *ρ_q_* and *μ_q_* represent the mass density (2.648 g/cm^3^) and shear modulus (2.947 × 10^11^ g·cm^−1^·s^−2^) of AT-cut quartz, respectively. Additionally, a control experiment was conducted using anti-AFP detection antibodies without WO_3_ tags under identical UV + AgNO_3_ conditions (Table S2). In this case, the resonance frequency shift (*Δf*) was 148 Hz at an AFP concentration of 10 ng/mL, confirming the critical role of WO_3_ in photocatalytic silver deposition and signal amplification. Meanwhile, the small residual decrease in resonance frequency after the final washing step in Figure 4 is attributed to hydration of the silver layer and residual ionic adsorption on the surface rather than true mass increase.

### 3.4. Improving AFP Detection Sensitivity Using WO_3_-Mediated Photocatalytic Silver Staining

**Figure 5:**
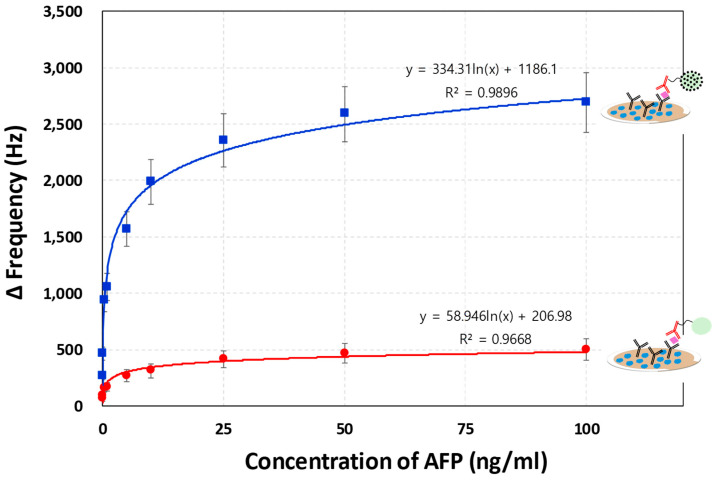
Resonance frequency shifts in the QCM immunosensor at various AFP concentrations (50 pg/mL–100 ng/mL). The red plot illustrates results from the sandwich immunoassay using WO_3_ nanoparticle-conjugated detection antibodies without signal amplification. The blue plot at the top shows the enhanced frequency shifts obtained through photocatalytic silver staining-based signal amplification.

We investigated the effect of varying concentrations of alpha-fetoprotein (AFP) ranging from 50 pg/mL to 100 ng/mL in AFP-free human serum on the resonance frequency shifts in a QCM sensor during a sandwich immunoassay followed by photocatalytic silver staining. For each AFP concentration, the average frequency shift was calculated from three independent experiments. As depicted in Figure 5, the resonance frequency shift increased logarithmically with increasing AFP concentration. At higher AFP concentrations, a greater amount of the target protein was captured by the immobilized paired antibodies, resulting in an increased accumulation of WO_3_ nanoparticles conjugated to the detection antibody on the QCM sensor surface. This increase in surface-bound nanoparticles contributed to the mass loading on the sensor and was further amplified by the enlargement of the WO_3_ nanoparticles via the photocatalytic silver deposition process. To isolate the mass-loading effect from other confounding factors, frequency shifts at each concentration were corrected by subtracting the blank values. This correction was essential to eliminate the influence of non-specific factors such as solution viscosity and temperature fluctuations, which were introduced during the silver staining process involving the addition of AgNO_3_ and UV irradiation. It is well known that in most QCM-based biomolecular detection systems, the thickness of the adsorbed biomolecular layer is typically much smaller than the viscous penetration depth in the liquid phase. Therefore, viscosity changes can significantly affect the resonance frequency. However, in this study, the presence of a signal amplification step involving photocatalytic nanoparticle growth necessitated rigorous blank subtraction to ensure that the observed frequency changes reflected only the mass-loading effect due to AFP binding.

The blue plot in Figure 5 illustrates the results of a WO_3_ nanoparticle-conjugated sandwich immunoassay without signal amplification, while the upper red plot shows the enhanced response achieved through photocatalytic silver staining-mediated signal amplification. As previously described, the silver staining process significantly contributed to the overall decrease in resonance frequency due to the increased mass loading and nanoparticle growth on the sensor surface. Signal amplification using photocatalytic silver staining (red plot) substantially improved the sensor’s sensitivity, achieving a limit of detection (LOD) for AFP of 43.7 pg/mL. In contrast, the LODs for the assays without amplification were 286 pg/mL (blue plot). In all cases, the LOD, calculated according to Shrivastava and Gupta [27], was defined as follows (Equation (2)):

(2) $$LOD={Mean}_{blank}+1.645\left({SD}_{blank}\right)+1.645\left({SD}_{low\;concentration\;sample}\right)$$

Owing to the enhanced signal intensity resulting from silver deposition, the detectable AFP concentration was markedly lowered, corresponding to an approximately 6.5-fold improvement in the limit of detection compared with the blue plot. A magnified inset of the low AFP concentration region (Figure S4) clearly demonstrates the distinct frequency shifts enabling precise LOD determination. Furthermore, the linear correlation (R^2^) between AFP concentration and resonance frequency improved with signal amplification. The correlation coefficient increased from 0.9668 (red plot) to 0.9896 (blue plot) with silver staining (Figure 5), indicating better linearity and quantitative reliability. The coefficient of variation (CV), a key factor for reproducibility, was 30.6% at the lowest AFP concentration (50 pg/mL) for the red plot. However, when the same assay was followed by photocatalytic silver staining, the CV values across all tested concentrations were reduced to below 13.6%, demonstrating significantly improved assay precision (Table S3). Further enhancement in the reproducibility at AFP concentrations below 5 ng/mL could be achieved by using monodisperse WO_3_ nanoparticles to minimize heterogeneity, enabling oriented antibody immobilization through protein A/G linkers, and incorporating automated fluidic control to reduce manual variability. Taken together, these findings indicate that the QCM-based AFP immunosensor employing WO_3_ nanoparticle-mediated photocatalytic silver staining provides a highly sensitive and reproducible platform, with an LOD of 43.7 pg/mL. This performance is comparable to, or better than, several recently reported nanomaterial-based AFP biosensors, while offering several distinct innovations. WO_3_ nanoparticles are employed for the first time as both a photocatalyst and an antibody carrier, simplifying the sensor design and enhancing photocatalytic efficiency. The UV-driven silver deposition proceeds without additional reducing agents, providing an environmentally friendly and well-controlled amplification strategy that significantly improves reproducibility (CV < 13.6%). Furthermore, the platform enables label-free, mass-sensitive, real-time detection, offering clear advantages over conventional ELISA or fluorescence assays. A comparative summary of LODs from various detection methods, including the present study, is provided in Table 1.

In summary, in addition to the effect of silver enhancement, both the average particle diameter and polydispersity index (PDI) of WO_3_ nanoparticles influence the uniformity of antibody binding, the available photocatalytic surface area, and light transmittance, which collectively determine the sensitivity and precision of AFP immunoassay. A narrower PDI ensures uniform photocatalytic activity and consistent Ag deposition, thereby improving reproducibility. Furthermore, controlling the morphology (e.g., nanorods or monodisperse spherical particles) and uniform size distribution of WO_3_ nanoparticles could further enhance the precision of the sensor in future studies.

### 3.5. Evaluation of QCM Sensor Performance Using Blind-Spiked AFP Samples

**Figure 6:**
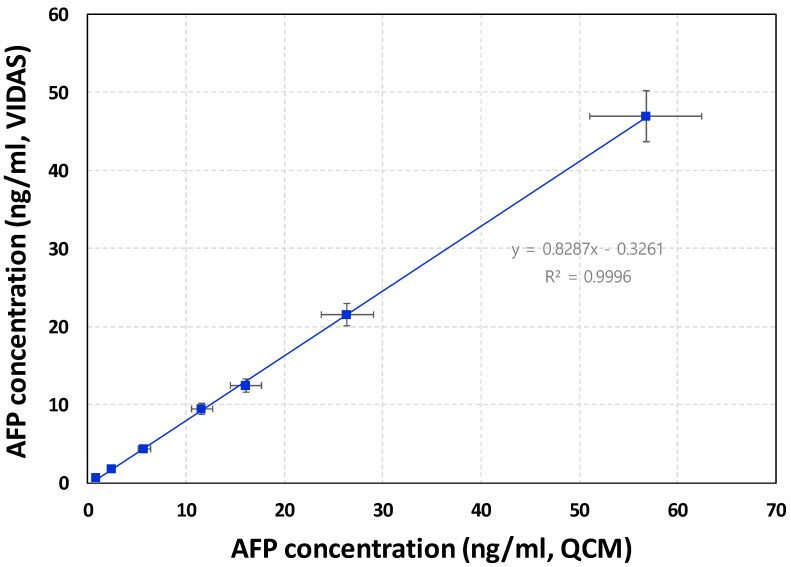
Correlation analysis between AFP concentrations measured by the developed QCM sensor system and the commercial VIDAS^®^ immunoassay for seven blind-spiked human serum samples. A strong agreement was observed between the two methods across the entire tested concentration range (0.5 ng/mL to 50 ng/mL), demonstrating the quantitative reliability of the QCM-based assay.

To validate the practical utility and analytical accuracy of our QCM-based immunoassay for AFP detection, a blind-spiked sample analysis was conducted using both our QCM sensor platform and a commercial reference system, the VIDAS^®^ immunoassay (bioMérieux, Marcy L’Étoile, France). Seven serum samples were independently prepared by spiking an undisclosed amount of AFP into AFP-free human serum (1 mL each). These blind samples were analyzed using the two systems, and the results were compared. In the QCM system, the resonance frequency shifts measured for the blind samples were converted into AFP concentrations using the standard calibration curve derived from the upper (blue) plot in Figure 5. The AFP concentrations obtained from both the QCM and VIDAS^®^ systems are presented in Table S4. For the QCM system, the mean value of three replicate measurements was used, while the VIDAS^®^ results represent the average of two replicates. Relative errors were calculated as the percentage difference between the QCM and VIDAS^®^ results, normalized to the VIDAS^®^ values. Although the AFP concentrations measured by the QCM system were higher overall compared to those from the clinically validated VIDAS^®^ system, the relative errors ranged between 20 and 35%. Despite these deviations, the two methods showed acceptable agreement across the entire AFP concentration range tested (0.5 ng/mL to 50 ng/mL), as illustrated in Figure 6. Deming regression analysis revealed a strong correlation between the two methods, with a correlation coefficient (R) of 0.9996 and a slope of 0.8287. This proportional bias (slope = 0.8287) is considered acceptable given that each assay is based on different analytical principles, calibration standards, and surface binding kinetics. Specifically, the QCM assay measures mass-induced resonance frequency shifts corresponding to total bound mass on the quartz surface, while the VIDAS^®^ system employs enzyme-linked fluorescence for signal transduction. Moreover, the QCM calibration curve was constructed using laboratory-prepared AFP-spiked serum samples, whereas VIDAS^®^ uses traceable proprietary calibrants aligned with clinical reference materials. These differences in signal generation mechanisms, calibration schemes, and antigen–antibody binding geometries can reasonably account for the observed systematic offset between the two measurement systems [39]. Additionally, the recovery rates of AFP spiked into human serum at three concentrations (5, 10, and 50 ng/mL) were assessed using the QCM immunoassay with photocatalytic silver staining. The observed recovery rates ranged from 116% to 123%, further supporting the quantitative performance of the developed assay (Table S5).

## 4. Conclusions

In this study, we successfully developed a sandwich immunoassay combined with a WO_3_-mediated photocatalytic silver staining technique for signal amplification, enabling highly sensitive and reproducible detection of AFP in human serum. The substantial enhancement in resonance frequency shift induced by the signal amplification markedly improved both the sensitivity and reproducibility of the assay. Given these advantages, the proposed QCM sensing platform holds great promise for broad applications in immunoassays and biomarker detection. To minimize the %RSD between the developed QCM system and the commercial platform, which represents the primary limitation of this study, several strategies are proposed, including optimization of Ag deposition kinetics, precise control of nanoparticle loading, and the incorporation of surface passivation layers to ensure uniform catalytic activity across different batches. Future work will focus on advancing the QCM biosensor system towards point-of-care testing by integrating automated fluidics and multiplexed target detection. In this context, a cartridge-based device incorporating a QCM sensor chip and a UV irradiation module capable of automated fluid control is envisioned, with materials such as quartz that allow efficient transmission of 365 nm UV light being essential for optimal performance.

## Figures and Tables

**Table 1:** Comparison of LODs in nanomaterial-assisted AFP detection with reported methods.

Detection Technique	Nanomaterials	LOD (pg/mL)	Linear Range (ng/mL)	Reference
Colorimetry Colorimetry Fluorescence Fluorescence Chemiluminescence Electrochemistry Electrochemistry SPR ^1^ SERS ^2^ ECL ^3^ QCM QCM	AuNP ^4^ ZnS-CdTe Carbon dots AuNP AuNP-Fe_3_O_4_ Ag/Bi_2_S_3_/TiO_2_ nanorod AuNPs–MXene composite Label-free AuNP Ru(bpy)_3_^2+^/β-cyclodextrin AuNP WO_3_/Ag	83 10 474 1.51 0.067 5.1 50 5.4 500 0.0011 56 43.7	10–300 0.05–12 10–100 0.01–0.8 0.0001–1.0 0.002–100 1.0–300 0.5–3.0 0.5–1000 0.00001–50 0.05–100 0.05–100	[28] [29] [30] [31] [32] [33] [34] [35] [36] [37] [38] This work

^1^ SPR: Surface plasmon resonance; ^2^ SERS: Surface Enhanced Raman Spectroscopy; ^3^ ECL: Electrochemiluminescence; ^4^ AuNP: Gold nanoparticle.

## Data Availability

Data are contained within the article.

## Supplementary Materials

The following supporting information can be downloaded at: https://www.mdpi.com/article/10.3390/bios15110728/s1, Figure S1. Concentration optimization of the immobilized capture antibody tested using a 9 MHz quartz crystal microbalance (QCM) resonator. When the probe concentration reaches 100 μg/mL, saturation begins to occur; Figure S2. The QCM sensor response upon the addition of 10 ng/mL AFP in human serum, captured by the antibody–WO_3_ conjugates, followed by photocatalytic silver staining, exhibits a marked frequency decrease and resistance increase, indicative of an increased effective mass on the QCM sensor surface; Figure S3. TEM images showing that excessive AgNO_3_ concentrations (20 mM) lead to aggregation and partial detachment of Ag nanoparticles from the WO_3_ surface after 5 min of UV irradiation; Figure S4. A “zoomed-in” inset of the AFP concentration range between 0.05–1 ng/mL. The red dashed plot illustrates results from the sandwich immunoassay using WO_3_ nanoparticle-conjugated detection antibodies without signal amplification. The blue dashed plot at the top shows the enhanced frequency shifts obtained through photocatalytic silver staining-based signal amplification; Table S1: The fluorescence intensity as a function of BSA concentration; Table S2: QCM signal variations upon UV irradiation under various experimental conditions; Table S3: QCM signal intensities (changes in resonance frequency) and % CV values; Table S4: Blind-spiked AFP sample tests using our QCM sensing system and the VIDAS^®^ immunoassay system; Table S5: Recovery rate of spiked AFP in human serum tested by our QCM sensing system.
