# Supplementary material for: Tungsten Oxide-Mediated Photocatalytic Silver Enhancement in a QCM Immunosensor for Alpha-Fetoprotein Detection
Source: Biosensors (Basel). 2025 Nov 2;15(11):728. doi: 10.3390/bios15110728 (PMC12650396; doi:10.3390/bios15110728)
Supplement: Supplementary file 1 [file biosensors-15-00728-s001.zip › biosensors-3902768-supplementary.pdf]

Tungsten Oxide-Mediated Photocatalytic Silver Enhancement in a QCM Immunosensor for Alpha-Fetoprotein Detection

Han Sol Kim, Yu Gyeong Cho and Soo Suk Lee*

Department of Pharmaceutical Engineering, Soonchunhyang University, Asan 31538, Republic of Korea
* Correspondence: sslee0810@sch.ac.kr; Tel.: +82-41-530-1394

1) Optimization of capture antibody concentration

Experimental results evaluating different concentrations of capture antibody have been provided.

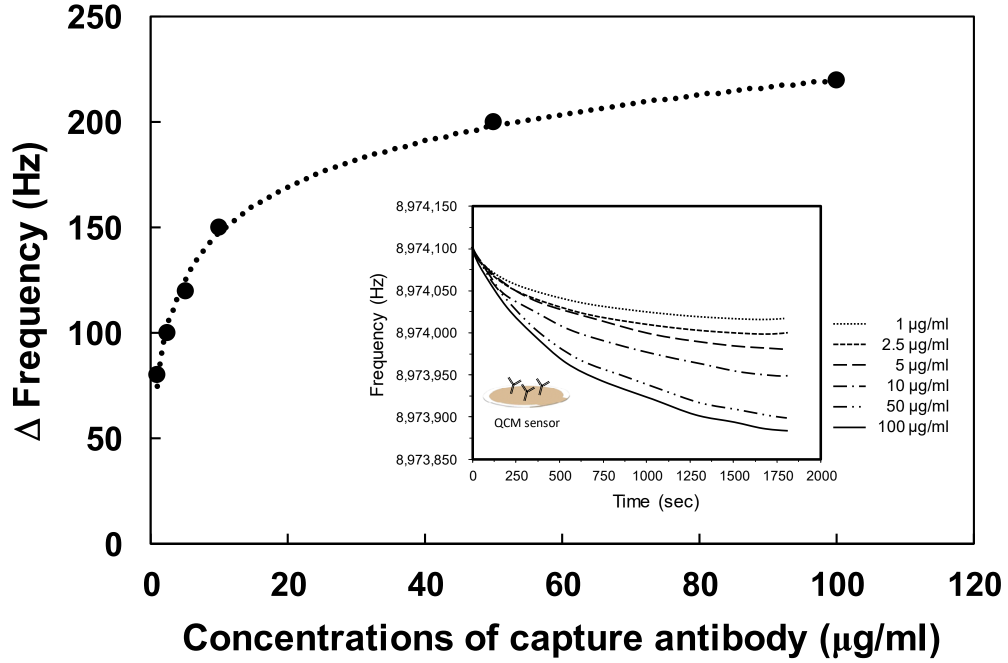

Figure S1. Concentration optimization of the immobilized capture antibody tested using a 9 MHz quartz crystal microbalance (QCM) resonator. When the probe concentration reaches 100 µg/mL, saturation begins to occur.

2) Optimization of BSA concentration for surface blocking

Experimental results evaluating different concentrations of BSA solution have been provided. The measurement was performed by immobilizing fluorescein-labeled antibodies onto the BSA-treated surface, followed by an initial washing step, after which the fluorescence intensity was recorded and

compared. By adjusting the amount of the BSA in the blocking buffer, it was found that the background signal decreased as the amount of BSA increased until a plateau appeared. Considering this trend, 3% BSA was selected as the optimal blocking concentration.

Table S1. The fluorescence intensity as a function of BSA concentration.

BSA concentration (%)	0.1	1	2	3	4	5
Fluorescence intensity (a.u, $\times 10^5$)	9.6 ± 1.6	8.7 ± 1.4	2.4 ± 0.3	1.6 ± 0.3	1.8 ± 0.4	1.7 ± 0.3

3) The changes in resonance resistance (A resonance resistance vs. time plot)

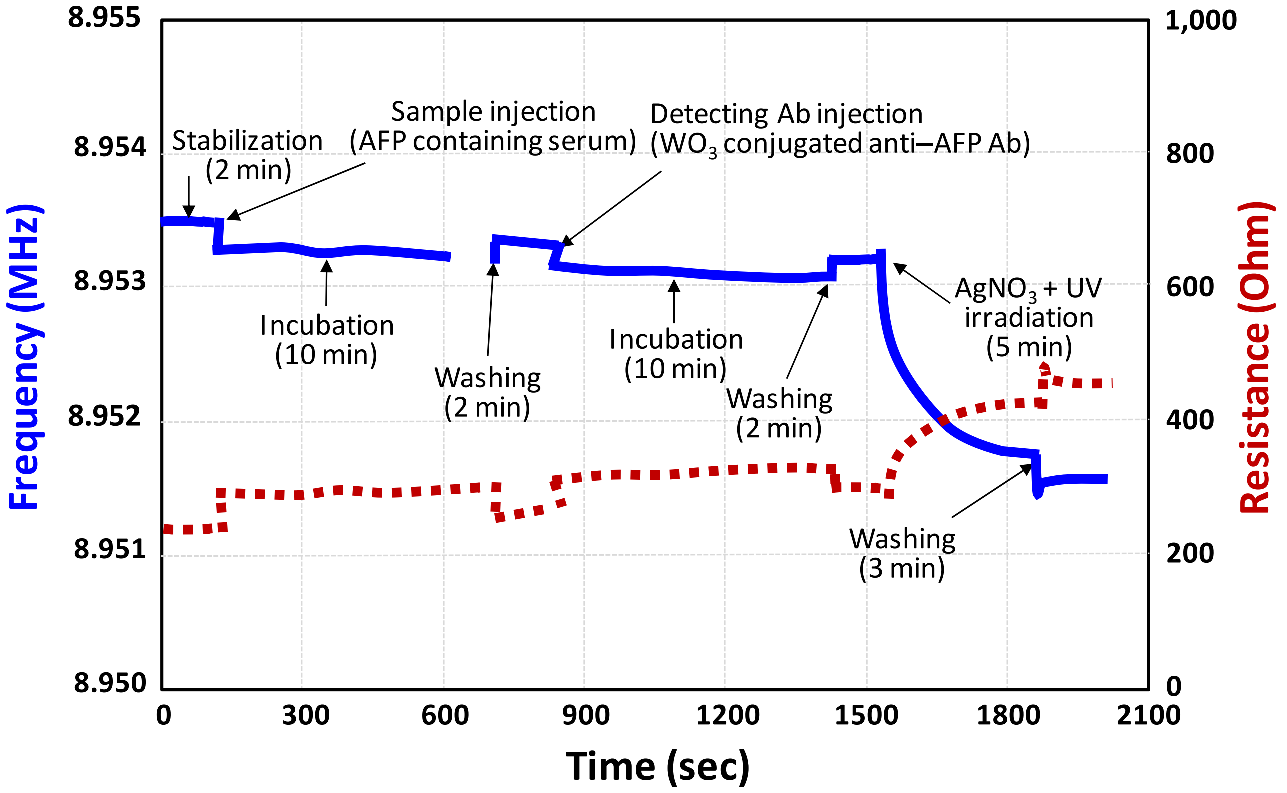

Figure S2. The QCM sensor response upon the addition of 10 ng/mL AFP in human serum, captured by the antibody–WO_3 conjugates, followed by photocatalytic silver staining, exhibits a marked frequency decrease and resistance increase, indicative of an increased effective mass on the QCM sensor surface.

4) TEM image showing the impact of excessive $AgNO_3$ concentration on the UV-assisted fabrication of WO_3/Ag hybrid nanostructures

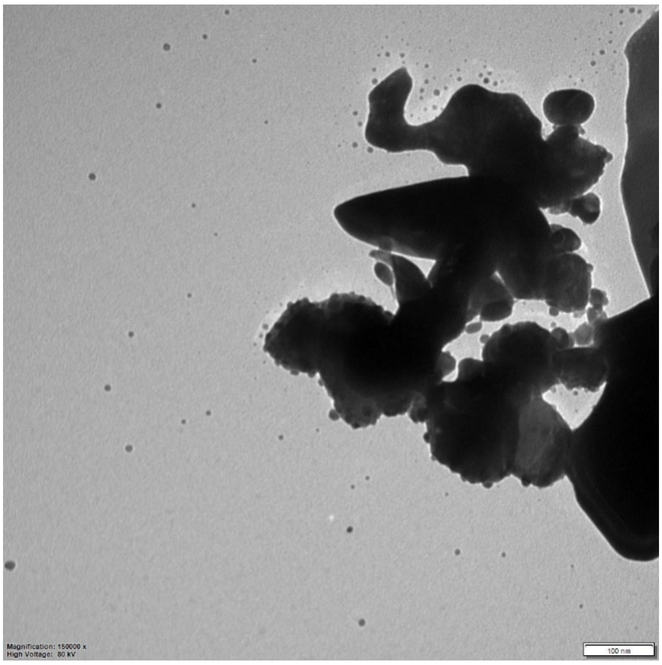

Figure S3. TEM images showing that excessive $AgNO_3$ concentrations (20 mM) lead to aggregation and partial detachment of Ag nanoparticles from the WO_3 surface after 5 min of UV irradiation.

5) The photocatalytic impact of WO_3 nanoparticles on the change in resonance frequencies after UV-assisted Ag staining on the QCM surface

In control experiments where $AgNO_3$ was subjected to UV irradiation on a bare QCM surface lacking WO_3, an antibody-functionalized surface, and a BSA-blocked surface, no appreciable variations in the QCM signal were observed.

Table S2. QCM signal variations upon UV irradiation under various experimental conditions.

	bare QCM surface (w/o WO_3)	Capture Antibody + BSA surface (w/o WO_3)	Sandwich Immunoassay (w/o WO_3)	Sandwich Immunoassay (with WO_3)
Δf (UV irradiation step)	37 Hz	33 Hz	38 Hz	2582 Hz

6) QCM signal intensities (changes in resonance frequency) and % CV values

The QCM signal intensities (changes in resonance frequency) and % CV values for the detection of AFP by WO_3 nanoparticle-conjugated sandwich immunoassay without signal amplification and including signal amplification by a photocatalytic silver staining reaction. Measurements were performed three times.

Table S3. QCM signal intensities (changes in resonance frequency) and % CV values.

Conc. (ng/mL)	WO_3-based immunoassay		With photocatalytic silver staining	
	Signal intensity (Hz)	CV (%)	Signal intensity (Hz)	CV (%)
0.05	62 ± 19	30.6	264 ± 36	13.6
0.1	92 ± 25	27.2	463 ± 57	12.3
0.5	154 ± 30	19.5	934 ± 98	10.5
1.0	167 ± 38	22.8	1,054 ± 118	11.2
5.0	265 ± 54	20.4	1,568 ± 156	10.9
10	312 ± 62	19.9	1,984 ± 198	10.0
25	415 ± 75	18.1	2,354 ± 236	10.0
50	467 ± 87	18.6	2,588 ± 241	9.3
100	498 ± 96	19.2	2,694 ± 265	9.8

7) Blind-spiked AFP sample tests

Table S4. Blind-spiked AFP sample tests using our QCM sensing system and the VIDAS® immunoassay system.

Sample number	QCM sensor system	VIDAS® immunoassay system	Relative error (%)*
Sample 1	0.831 ± 0.093 ng/mL	0.627 ± 0.048 ng/mL	32.5
Sample 2	2.40 ± 0.279 ng/mL	1.86 ± 0.153 ng/mL	29.0
Sample 3	5.76 ± 0.65 ng/mL	4.29 ± 0.34 ng/mL	34.3
Sample 4	11.6 ± 1.09 ng/mL	9.49 ± 0.69 ng/mL	22.2
Sample 5	16.1 ± 1.58 ng/mL	12.4 ± 0.86 ng/mL	29.8
Sample 6	26.4 ± 2.67 ng/mL	21.5 ± 1.38 ng/mL	22.8
Sample 7	56.8 ± 5.68 ng/mL	46.9 ± 3.26 ng/mL	21.1

* Relative error (%) = (VIDAS–QCM)/VIDAS × 100

8) Recovery rate of spiked AFP in human serum

Table S5. Recovery rate of spiked AFP in human serum tested by our QCM sensing system.

Samples[1]	Added (ng/mL)	Found (ng/mL)	Recovery (%)	RSD (n = 3) (%)
1	5.0	5.8 ± 0.89	116	15.3
2	10.0	12.1 ± 1.80	121	14.9
3	50.0	61.4 ± 8.84	123	14.4

9) A "zoomed-in" inset of the AFP concentration range between 0.05–1 ng/mL

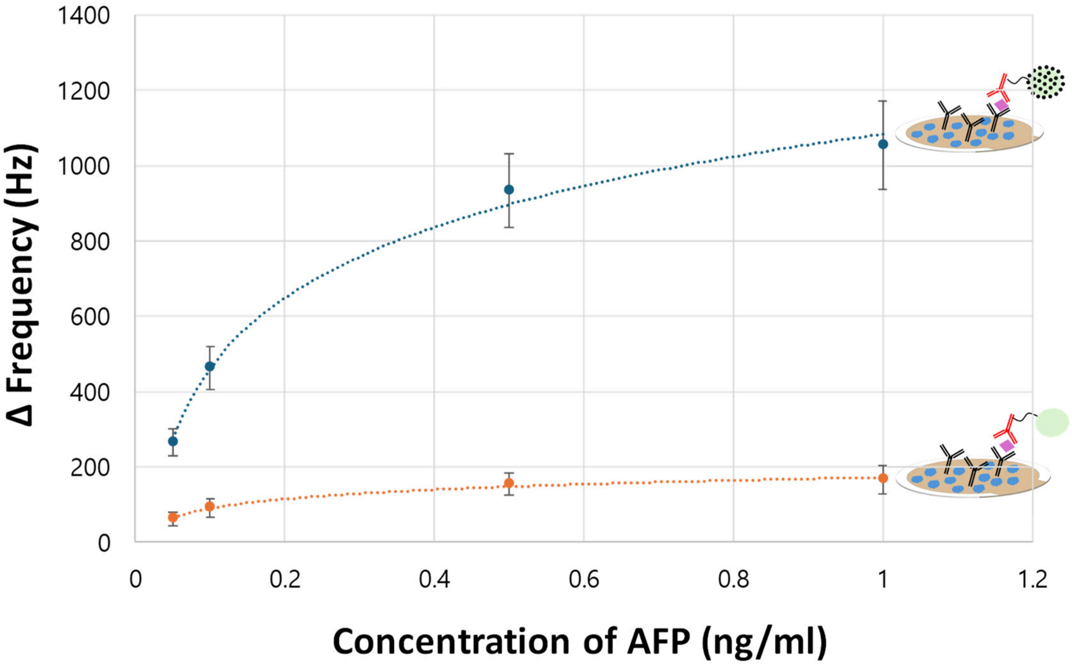

Figure S4. A "zoomed-in" inset of the AFP concentration range between 0.05–1 ng/mL. The red dashed plot illustrates results from the sandwich immunoassay using WO_3 nanoparticle-conjugated detection antibodies without signal amplification. The blue dashed plot at the top shows the enhanced frequency shifts obtained through photocatalytic silver staining-based signal amplification.
